# Co-Colonization by Multiple Multidrug-Resistant Organisms in a Long-Term Neurorehabilitation Setting: A Case Report

**DOI:** 10.3390/microorganisms14081731

**Published:** 2026-08-07

**Authors:** Camilla Grifoni, Chiara Bonaiuto, Nicla Giovacchini, Marco Coppi, Alberto Antonelli, Simona Pollini, Roberto Pupillo, Gian Maria Rossolini

**Affiliations:** 1 Severe Brain Injuries Unit, IRCCS Don Gnocchi Foundation, 50143 Florence, Italy; camilla.grifoni@unifi.it; 2 NARR Joint Laboratory, University of Florence, IRCCS Don Gnocchi Foundation, 50143 Florence, Italy; marco.coppi@unifi.it (M.C.); alberto.antonelli@unifi.it (A.A.); simona.pollini@unifi.it (S.P.); 3 Department of Experimental and Clinical Medicine, University of Florence, 50134 Florence, Italy; chiara.bonaiuto@unifi.it (C.B.); nicla.giovacchini@unifi.it (N.G.); 4 Microbiology and Virology Unit, Careggi University Hospital, 50134 Florence, Italy; 5 Medical Direction, IRCCS Don Gnocchi Foundation, 50143 Florence, Italy; rpupillo@dongnocchi.it

**Keywords:** active surveillance, multidrug-resistant organisms, long-term care rehabilitation hospitals, neurorehabilitation units, case report

## Abstract

Neurorehabilitation units (NRUs) are long-term care settings associated with multiple risk factors for carriage and infection by multidrug-resistant organisms (MDROs), including prolonged hospitalization, the use of invasive medical devices, high antibiotic exposure, and multiple comorbidities. We report the case of a patient admitted to an NRU for rehabilitation after cerebral hemorrhage who developed complex co-colonization by several MDROs, including *Pseudomonas aeruginosa* producing either FIM or VIM metallo-β-lactamases, *Acinetobacter baumannii* producing OXA-23 serine-carbapenemase, *Klebsiella pneumoniae* producing either KPC or OXA-48 serine-carbapenemases or both, *Proteus mirabilis* producing VEB extended-spectrum β-lactamase, and vancomycin-resistant *Enterococcus faecium* of the VanA type. During the 22-month hospitalization, the patient experienced several infection episodes, some of which were apparently related with colonizing MDROs. This case highlights that co-colonization by multiple MDROs may occur in patients admitted to NRUs in endemic settings, and emphasizes the importance of implementing targeted infection prevention and control strategies in these healthcare environments.

## 1. Introduction

The increasing incidence of infections caused by multidrug-resistant organisms (MDROs) represents a major public health challenge on a global scale [1]. Healthcare-associated infections (HAIs) caused by these pathogens are among the most relevant complications of hospital care and are associated with increased morbidity, mortality, prolonged hospitalization, and higher healthcare-associated costs. The high prevalence of vulnerable patients, extensive exposure to antimicrobial agents, frequent use of invasive medical devices, and the complexity of diagnostic and therapeutic procedures contribute to the selection and transmission of MDROs in acute-care hospitals (ACHs) [2]. However, the epidemiology of MDROs extends beyond ACHs, involving a network of interconnected healthcare settings. In fact, patients discharged from ACHs to long-term rehabilitation hospitals (LTRHs) may carry MDROs as colonizers acquired during previous hospitalizations, whereas in LTRHs, multiple risk factors—including prolonged hospitalization, the use of invasive medical devices, high antibiotic exposure, multiple comorbidities, and involvement in rehabilitation programs in shared environments—may facilitate further selection and dissemination of MDROs [3]. Moreover, subsequent readmissions of some of these patients to ACHs, either due to clinical deterioration or complications, may contribute to further exposure to resistant pathogens. This continuous exchange of patients between different healthcare settings, commonly referred to as the “revolving door” phenomenon, represents an important mechanism contributing to the persistence and spread of MDROs in healthcare settings [3].

In LTRHs, neurorehabilitation units (NRUs) are clinical contexts affected by a notably high risk for infections due to the increase in several risk factors, including: (a) the provenance of patients from intensive-care units of ACHs; (b) lengths of stay up to several months; (c) the invariable presence of invasive devices, such as urinary catheters, gastrostomy tubes, central venous lines, tracheostomy cannulas, and mechanical ventilation; (d) frequent occurrences of complex wounds and/or pressure ulcers; and (e) the difficulties in implementation of strict infection prevention and control (IPC) measures due to motor and cognitive limitations of patients, requiring a high level of care by caregivers [4]. Moreover, recent reports indicate that cerebrovascular events may predispose individuals to an increased risk of infection by reducing immune system protection. This increased susceptibility is not solely attributable to clinical factors commonly associated with severe acquired brain injury management, such as hospitalization, invasive procedures, dysphagia, aspiration, and bladder dysfunction, but also results from an intrinsic alteration of the neuro-immune interaction induced by brain injury. Acute cerebrovascular damage can trigger a maladaptive immunosuppressive response, characterized by a downregulation of both innate and adaptive immunity, a condition known as stroke-induced immunodepression or CNS injury-induced immunodepression (CIDS). Although this response may initially represent a protective mechanism aimed at limiting excessive inflammation after brain injury, its persistence can impair host defense mechanisms, creating a favorable environment for opportunistic infections and negatively affecting neurological recovery and clinical outcomes [5]. Consequently, in areas of high endemicity for MDROs, the NRUs of LTRHs can be a major reservoir for these pathogens, namely methicillin-resistant *Staphylococcus aureus* (MRSA), vancomycin-resistant enterococci (VRE), extended-spectrum beta-lactamase (ESBL)-producing Enterobacterales, carbapenemase-producing Enterobacterales (CPE), carbapenem-resistant *Acinetobacter baumannii* (CRAb) and difficult-to-treat resistant *Pseudomonas aeruginosa* (DTR-Pa) [3].

In this report, we describe the case of a patient from an NRU that exhibited simultaneous colonization by several different MDROs, some of which were apparently associated with infections occurred during the prolonged hospital stay, highlighting the complexity that the MDRO challenge can reach in these settings. MDRO colonization was defined as the isolation of an MDRO from surveillance cultures in the absence of clinical signs or symptoms of infection. Infectious episodes were classified according to clinical and microbiological findings, using the European Centre for Disease Prevention and Control (ECDC) case definitions for healthcare-associated infections in long-term care facilities (HALT-3 protocol) [6]. For urinary tract infections (UTIs) and lower respiratory tract infections (LRTIs), diagnoses required the presence of the clinical criteria defined by the ECDC case definitions together with microbiological findings. Bloodstream infections (BSIs) were considered confirmed when clinically significant pathogens were isolated from at least two sets of blood cultures.

## 2. Case Presentation

The case patient, who was in their late seventies, had been transferred to the intensive-care NRU of an LTRH for neurorehabilitation following cerebral hemorrhage that had required decompressive craniectomy after endovascular treatment. The LTRH was located in an area of high resistance endemicity in Central Italy, and deals with the rehabilitation of patients requiring variable intensities of care, including those affected by severe brain injuries. The provenance ACH was also located in an area of high resistance endemicity in Southern Italy.

Upon admission, the patient was comatose, on mechanical ventilation via tracheostomy, and on artificial nutrition via percutaneous endoscopic gastrostomy (PEG), with presence of pressure ulcers at the occipital and sacral levels. Screening for CPE carriage at admission, performed as a routine practice on admission to the LTRH when no information is provided at patient handover, revealed rectal carriage of KPC-producing *Klebsiella pneumoniae*.

**Figure 1:**
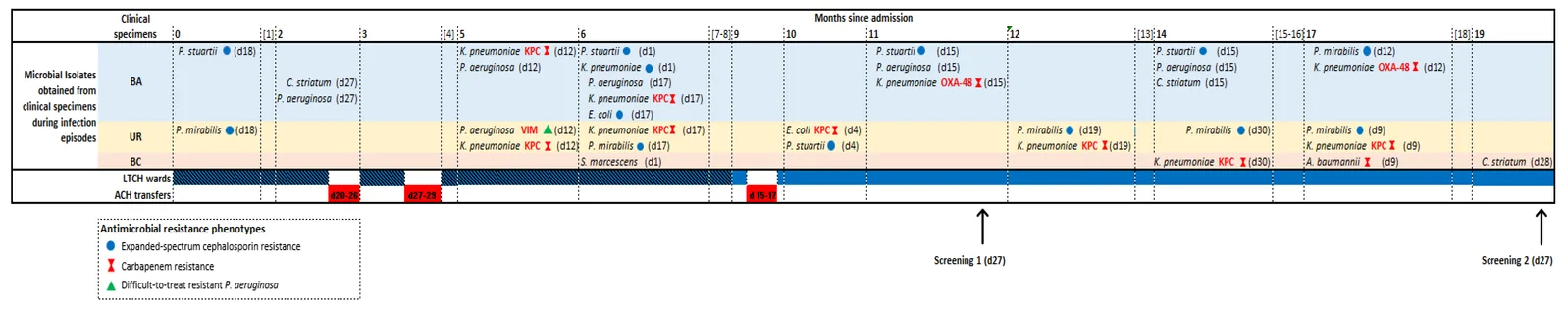
Temporal diagram describing the stay of the case patient at the LTRH (blue line), including the intensive (striped) and intermediate (plain) neurorehabilitation units where the patient received care and occasional transfers to the nearby ACH (red), as required for various reasons. The infectious episodes diagnosed during the hospital stay, along with microbial pathogens isolated from bronchial aspirate (BA), urine (UR), or blood (BC) associated with the infectious episodes. For each episode, timing is reported in months (m) and days (d) since admission, with m0d0 corresponding to the date of admission. Conventional diagnostic microbiology was outsourced to a private provider, and the results on microbial isolates, resistance phenotypes, and resistance mechanisms were based on data returned from the external provider and stored in the clinical record. The relevant resistance profiles of clinical isolates (namely resistance to expanded-spectrum cephalosporins for Enterobacterales, resistance to carbapenems for Gram-negatives, and difficult-to-treat resistance for *P. aeruginosa*) and the presence of resistance mechanisms (when available) are reported for clinical isolates. The dates of the two broad screenings for MDRO carriage (at m11d27 and m19d27) are also indicated.

During the almost two-year stay at the LTRH, the patient was initially kept for eight months in the intensive-care NRU before being transferred to the intermediate-intensity NRU, and experienced several episodes of infections (UTIs, LRTIs, and BSIs), which were diagnosed as described above and associated with the isolation of several different microbial pathogens (Figure 1). The infectious episodes required several cycles of antimicrobial chemotherapy and, in two cases, the transfer to a nearby ACH for short periods (one day and six days each). Additional transfers to the same ACH were consequent to the need for routine PEG replacements (performed at least every 6 months). Exitus occurred after 22 months of stay in the LTRH and was attributed to heart failure (Figure 1).

After 11 months of hospital stay, as part of an epidemiological investigation related to a case of fatal infection by carbapenemase-producing *P. aeruginosa* observed in another inpatient who had shared the same ward, the patient underwent comprehensive screening for carriage of MDROs, and screening was repeated again after eight months (Figure 1). Screening for MDRO carriage was initially performed on DNA extracted from rectal and pharyngeal swabs (Fecal Swab and ESwab systems; Copan, Brescia, Italy) using the STARMag Universal Cartridge kit (Seegene, Seoul, Republic of Korea) and a real-time PCR targeting several resistance genes, including *bla*_KPC_, *bla*_VIM_, *bla*_OXA-48_, *bla*_NDM_, *bla*_IMP_, *bla*_FIM_, *bla*_OXA-23_, *bla*_OXA-24_, *bla*_OXA-58_, *ISAba1*-*bla*_OXA-51_, *bla*_CTX-M_, *bla*_GES_, *bla*_PER_, *bla*_VEB_, *mecA/C*, and *vanA/B*, as described previously [7,8,9]. Specimens that were positive for one or more targets were then cultured on selective media (ChromID CARBA SMART, ChromID VRE, ChromID ESBL, bioMérieux, Marcy-l’Étoile, France) and also on Mueller–Hinton agar (Becton Dickinson, Franklin Lakes, NJ, USA) to isolate resistant strains carrying the corresponding resistance determinants. Identification of MDRO isolates was performed by MALDI-ToF mass spectrometry (MALDI Biotyper^®^ sirius System; Bruker, Berlin, Germany), and the presence of resistance determinants was confirmed by real-time PCR. The results of molecular screening revealed the presence of several different resistance determinants from both rectal and pharyngeal swabs, and cultural analysis yielded isolates of various species carrying the resistance determinants (Table 1). Altogether, the first screening revealed the presence of Gram-negative bacilli of different species (*K. pneumoniae*, *E. coli*, *P. aeruginosa*, and *A. baumannii* complex) that were positive for various serine- and metallo-carbapenemases at either rectal or respiratory levels or both, while the subsequent screening (performed after 19 months of hospital stay) identified not just the presence of most of the previously detected MDROs but also revealed the presence of carbapenemase- or ESBL-producing enterics, as well as *vanA*-positive VRE from rectal swabs (Table 1).

## 3. Discussion and Conclusions

This clinical case highlights the complexity of the MDRO scenario encountered in a patient with severe brain injury admitted for almost two years in the NRU wards of an LTRH from an area of high resistance endemicity, raising a number of issues for discussion.

Active surveillance is part of the IPC bundles for contrasting the transmission of MDROs in healthcare settings, and has been variably recommended in different settings and for different MDROs. In the LTRH where this case was observed, an active surveillance program for CPE in rectal swabs has been enforced for all new admissions when information on CPE carriage is not already available from the referring hospitals at handover, in consideration of the local epidemiology of resistance and in agreement with the recommendations of the GLISTer group of the Italian Association of Clinical Microbiologists (AMCLI) [10]. Following this practice, the case patient was found to be colonized by KPC-positive *K. pneumoniae* at admission, which was apparently involved in recurrent UTIs and possibly also LRTIs during the hospital stay (Figure 1). However, a broader screening for MDRO carriage, performed on both rectal and pharyngeal swabs 11 and 19 months after admission, revealed a much more complex pattern of MDRO carriage, including additional CPE, MBL-producing *P. aeruginosa*, CRAb, ESBL-producing *P. mirabilis*, and VRE; of which, some were also apparently involved in infections observed during the long hospital stay (Figure 1). Of these MDROs, KPC-positive *E. coli*, OXA-48-positive *K. pneumoniae*, and KPC+OXA-48-positive *K. pneumoniae* were apparently acquired by the patient after admission to the LTRH, as they were not detected following screening for CPE performed at admission. Concerning the other MDROs detected by broader screening, it is not possible to know whether they were already present at admission or were acquired during the hospital stay or following the short occasional transfers to the nearby ACH, as active surveillance for MDRO carriage other than CPE is not routinely performed at admission. Moreover, since broad MDRO screening was only performed after 11 and 19 months since admission, it was not possible to determine when most organisms were acquired, nor whether repeated detection represents persistence or recurrent acquisitions of MDROs.

Being a known CPE carrier at admission, the patient was subjected to routine IPC practices adopted for these cases, and these were likely able to limit the risk of dissemination of other MDROs. Nevertheless, during hospitalization, FIM-1-positive *P. aeruginosa* carried by the patient was apparently transmitted to another patient who had shared the same ward [11]. The present findings therefore raise questions about the potential usefulness of broadening screening for other MDROs and in other samples (e.g., pharyngeal swabs) and of repeating screening periodically, as well as for among CPE-positive patients.

During the long hospital stay, the patient experienced several infectious episodes, including four episodes of BSI with known bacterial causes and several episodes of UTIs and LRTIs associated with the isolation of potential causative pathogens (although the roles of some of these as colonizers could not be excluded). The bacterial pathogens involved in BSIs and associated with UTIs and LRTIs likely corresponded, at least in some cases, to the colonizing MDROs, as suggested by their reported resistance profiles and mechanisms. However, clinical isolates were not available for comparison with colonizing pathogens since routine diagnostic microbiology was outsourced to a private laboratory, which represents a limitation of this study. Therefore, the involvement of colonizing MDROs as the causes of infections remains hypothetical, as this was only based on species identification and the resistance phenotypes and mechanisms reported by the external laboratory.

Altogether, the present findings have highlighted the outstanding complexity of the profile of colonizing MDROs that can be observed in patients from NRU settings of LTRHs in areas of high endemicity for antimicrobial resistance, emphasizing the need for improvement of surveillance and IPC practices in similar settings where adoption of strategies tailored to rehabilitation dynamics would deserve further discussion. Moreover, the overall clinical history of this case suggested a variable contribution of some of the colonizing MDROs to infectious episodes recurrently observed, underscoring the complexity of relationships between carriage and infection that should be considered when selecting empirical antimicrobial chemotherapy in these settings for patients colonized by MDROs.

Future perspectives should not only focus on improved surveillance and antimicrobial stewardship strategies but should also consider the importance of innovative approaches to counteract MDROs. In particular, modified antibiotics that exploit bacterial metallophores to enhance entry into Gram-negative pathogens (the so-called Trojan horse strategy) are currently in development, with one landmark representative, cefiderocol, already introduced in clinical practice [12,13,14]. Another promising approach involves lytic bacteriophages targeting specific MDROs, such as high-risk clones of *Klebsiella pneumoniae*, which could complement conventional antimicrobial treatments [15,16]. Furthermore, MDRO colonization may represent a risk factor by itself for the acquisition of additional MDROs, potentially creating a complex microbial ecology that promotes co-colonization [10,17]. An integrated antimicrobial and diagnostic stewardship program, tailored to each rehabilitation setting and coordinated by a multidisciplinary team of infection disease specialist, clinical microbiologists, and neurorehabilitation specialists, could improve the early identification, management, and control of MDROs. Furthermore, the particular conditions of patients with neurological injuries warrant further investigation into the role of interactions between the nervous system, gut, and host immune response in influencing MDRO colonization dynamics and infection risks.

## Figures and Tables

**Table 1:** MDROs detected following the two screenings for MDRO carriage, performed using rectal and pharyngeal swabs. Methods for bacterial identification and characterization of resistance mechanisms are described in the text.

Specimen	MDRO Isolates from Screening 1	MDRO Isolates from Screening 2
**Rectal swab**	*K. pneumoniae* KPC FIDG-26375	*K. pneumoniae* KPC FIDG-4402
	*E. coli* KPC FIDG-26376	*E. coli* KPC FIDG-4401
	*K. pneumoniae* OXA-48 FIDG-26374	*K. pneumoniae* KPC+OXA-48 FIDG-4403
	*A. baumannii* OXA-23 FIDG-27249	*A. baumannii* OXA-23 FIDG-4405
	*P. aeruginosa* VIM FIDG-26381	*P. mirabilis* VEB FIDG-4404
		*E. faecium* VanA FIDG-4407
**Pharyngeal swab**	*K. pneumoniae* OXA-48 FIDG-26379	*K. pneumoniae* OXA-48 FIDG-4410
	*K. pneumoniae* KPC FIDG-26378	*K. pneumoniae* KPC FIDG-4411
	*P. aeruginosa* VIM FIDG-26380	*P. aeruginosa* VIM FIDG-4409
	*P. aeruginosa* FIM-1 FIDG-26323	*P. aeruginosa* FIM FIDG-4408

## Data Availability

The original contributions presented in this study are included in the article. Further inquiries can be directed to the corresponding author.

## Abbreviations

The following abbreviations are used in this manuscript:

ACH	Acute-care hospital
BSI	Bloodstream infection
CPE	Carbapenemase-producing *Enterobacterales*
CRAb	Carbapenem-resistant *Acinetobacter baumannii*
DTR-Pa	Difficult-to-treat resistant *Pseudomonas aeruginosa*
ESBL	Extended-spectrum beta-lactamase
IPC	Infection prevention and control
LRTI	Lower respiratory tract infection
LTRH	Long-term rehabilitation hospital
MALDI-ToF	Matrix-assisted laser desorption ionization time of flight
MDRO	Multidrug-resistant organism
NRU	Neurorehabilitation unit
PCR	Polymerase chain reaction
PEG	Percutaneous endoscopic gastrostomy
UTI	Urinary tract infection
VRE	Vancomycin-resistant Enterococcus
